# Mental health literacy and seeking for professional help among secondary school students in Slovakia: a brief report

**DOI:** 10.3389/fpubh.2024.1333216

**Published:** 2024-01-30

**Authors:** Lenka Sokolová

**Affiliations:** ^1^Institute of Applied Psychology, Faculty of Social and Economic Sciences, Comenius University Bratislava, Bratislava, Slovakia; ^2^European Federation of Psychology Teachers’ Associations (EFPTA), Brussels, Belgium

**Keywords:** mental health literacy, help-seeking, secondary school, vocational education, psychology

## Abstract

Secondary school students are at risk for mental health problems, especially nowadays, when we face an increase in mental health issues among adolescents and the general population. Mental health literacy (MHL) and help-seeking behavior are considered protective factors; however, we lack data on the levels of MHL in secondary school students and how MHL is developed in schools. This correlational and comparative study was designed to investigate mental health literacy (MHL) and help-seeking behavior among secondary school students in Slovakia. A convenient sample of 250 Slovak secondary school students responded to an anonymous online survey consisting of two scales (Mental Health Literacy Scale and Self-stigma of Seeking Help). Significant differences in mental health literacy (*t(248)* = 5.56; *p* ≤ 0.0) and stigma of seeking help (*t(248)* = −4.33; *p* ≤ 0.01) were observed between students in general and vocational secondary school. Students in general secondary school who attended optional psychology courses had the highest scores in mental health literacy (*U* = 987; *p* = 0.003). These preliminary findings showed that secondary students with a higher level of mental health literacy reported a lower level of self-stigmatization related to seeking professional mental health help (*r* = −0.339; *p* ≤ 0.01). Implementing mental health education into secondary school curricula can potentially help increase mental health literacy and decrease self-stigma of seeking help.

## 1 Introduction

Mental health literacy (MHL) covers a variety of knowledge, attitudes, and skills related to mental health, including, e.g., knowledge about mental health and mental disorders, attitudes, and skills related to the prevention of mental health problems, self-help, self-care, or help-seeking behaviors (1–3). MHL as a construct is based on the concept of health literacy (4) and that is why it is often described and analyzed in connection with general health literacy (5). Another closely related construct is psychological literacy (6, 7). Similarly to psychological literacy, the level of MHL is discussed within the professional community and the general population. At the community level, MHL is related to the promotion of mental health care and healthcare in general (8), the promotion of help-seeking efficacy (9), help-seeking behavior (10), and self-stigmatization (11). While the first conceptualization of mental health literacy emphasized mainly the knowledge needed for the identification of mental disorders (12), the current approaches promote positive mental health literacy, which refers to an individual’s “awareness of how to achieve and maintain good mental health” (8).

Adolescence is often described as a formative period and at the same time, the period of high risk of mental health problems, according to the World Health Organization (13) “one in seven 10-19-year-olds experiences a mental disorder, accounting for 13% of the global burden of disease in this age group.” The findings of the recent international Health Behavior in School-aged Children (HBSC) survey highlighted the fact that well-being and mental health declined between the surveys in 2017/2018 and 2021/2022. One third of adolescents (aged 11–15) from 44 countries reported feeling lonely in the last year, nervous, or irritable more than once a week in the last six months (14). In recent years, adolescents have faced many challenges, including the COVID-19 pandemic, war conflicts, challenges in their digital lives, environmental threats, or social and political changes on national and local levels. These appear to have a negative impact on their well-being and mental health. The HBSC teams (14, 15) emphasized the need to monitor the mental health and well-being of adolescents, promote good mental health, and prevent mental health problems. Schools have been identified as key players in the delivery of mental health programs and the support of adolescent mental health and well-being.

Various providers offer mental health programs to promote and support mental health in adolescence (16, 17) and young adulthood (18), including general school-based interventions. These may improve student well-being and mental health and equip them with self-care, self-help, and help-seeking skills for both personal life and transition to higher education and professional life. Schools provide these interventions as short-term programs and long-term whole-school approaches (e.g., (16)), or these topics are included in the school curriculum as part of compulsory or optional subjects (19). In many countries, psychology is one of the subjects that covers topics of self-knowledge and understanding mental health (20). Psychology as an optional subject is taught in both general and vocational secondary education and the introduction to psychology is also included in the higher education training of various non-psychological professionals (21). The proportion of mental health content included in curricula differs between countries, study programs, and individual schools. Among others, positive mental health literacy and education-based programs are likely to be effective in improving MHL among adolescents (22, 23). General secondary schools offer more academic subjects, and vocational schools focus on practical skills, this often makes it difficult to develop metacognitive competence, critical thinking, and other literacies in vocational training (24, 25).

In Slovakia, adolescents can choose their future study path approximately at the age of eleven or at the age of fifteen. They continue their studies either at a general secondary school (grammar school), which is mainly aimed at preparing students for further studies in higher education, or they choose a vocational secondary school. Vocational schools train students for a variety of professions, including, e.g., healthcare, social and administrative staff, or technical professions. Secondary school curricula do not include specific subjects focused on mental health topics; however, mental health knowledge and skills are partially included in other subjects (e.g., psychology, ethics, biology, etc.). Psychology courses in both grammar and vocational schools usually cover a general overview of mental disorders and illnesses, stress, coping, and psychohygiene (20), however, psychology courses in grammar schools are more academically focused. The provision varies between schools and does not seem to be adequate.

This study aims to explore MHL and help-seeking behavior among secondary school students and to compare MHL levels between general and vocational school students. Studying mental health literacy in adolescents can contribute to the development of comprehensive educational programs to equip adolescents with the knowledge and skills necessary to maintain their own mental well-being and support others. Based on previous research, we hypothesize a negative correlation between MHL and self-stigmatization of help-seeking behavior and higher levels of MHL in grammar school students.

## 2 Method

### 2.1 Study design, setting, and sample size

The study was designed to assess the observed differences in MHL and self-stigmatization of help-seeking behavior between general and vocational school students and to explore the correlation between MHL and self-stigmatization of help-seeking behavior. An *a priori* power analysis was conducted using G*Power version 3.1.9.7 (26) for the estimation of the sample size, with a medium effect size, the minimum sample size needed was *N* = 176 for the comparison of two means (t-test). The inclusion criteria for this study were being a secondary school student in Slovakia, a minimum of 18 years old. Participants were recruited through their teachers (members of the Slovak psychology teacher network). The researcher contacted the teachers with the research information and invitation. Teachers then shared the link to the online survey with their students, the participation was voluntary and anonymous. Fifty percent of the secondary schools in Slovakia were contacted in all regions of the country and the data was collected from January to July 2023.

### 2.2 Participants

Participants in this study were Slovak students (*N* = 250; *M_age_* = 18.6; *SD* = 1.55), at the time of data collection they attended or currently finished their secondary school education. Seventy-seven students (31%) attended general secondary school (equal to grammar school/gymnasium) and the rest of the sample (*n* = 173; 69%) attended vocational secondary school (vocational study programs focus on professional training). Most of the vocational school students (*n* = 162, 94%) and 40% (*n* = 30) of the grammar school had at least one psychology course included in their curriculum.

### 2.3 Research ethics

The informed consent and the information about the research and its purposes were included as the first part of the questionnaire. Participants completed a fully anonymous online questionnaire voluntarily and without any reward or compensation. The last part of the questionnaire contained a message with useful contacts in case of mental health problems.

### 2.4 Instruments

To assess mental health literacy and help-seeking attitudes among secondary school students, we used two instruments. Both were translated from English into Slovak language using a back translation procedure. After the translation, verbal probing was used with two participants to verify their understanding of the individual items.

*Mental Health Literacy Scale - MHLS* (27). MHLS consists of 35 items that cover several areas of mental health literacy: the ability to recognize disorders and risk factors, the knowledge of where to seek information, the knowledge of available self-treatment and professional help, and attitudes that promote recognition or appropriate help-seeking behavior (27). The authors reported good psychometric properties of the scale and recommended using it as a one-dimensional instrument with a maximum score of 160 and a minimum of 35. The scale adequately discriminates the general population and mental health professionals or individuals with a history of mental illness (27). Based on the recommendation of the authors, we used the scale as unidimensional with good internal consistency (*α* = 0.82).

*Self-stigma of Seeking Help - SSOSH* (11). SSOSH is a measure consisting of 10 items evaluating different aspects of professional help-seeking behavior and related attitudes. The authors reported excellent internal consistency of the scale (*α* = 0.91) and suggested using it as a unidimensional scale measuring a single construct with a maximum score of 50 and a minimum of 10 (11). In our sample, the scale had adequate internal consistency (*α* = 0.72).

Data were analyzed using descriptive statistics, comparison of the means of two groups (t-test and U-test), and Pearson correlation coefficient.

## 3 Results

The mean score for MHLS in our sample was 120.54 (*SD* = 12.06; *min* = 90; *max* = 152; *skewness* = 0.45; *kurtosis* = 0.35). The mean score for the SSOSH was 23.84 (*SD* = 5.91; *min* = 10; *max* = 46; *skewness* = −0.11; *kurtosis* = −0.41). There were no missing data on both scales. Correlation analysis revealed a hypothesized negative significant correlation between perceived stigma of seeking professional help and the mental health literacy score (*r* = −0.339; *p* ≤ 0.01), which means that students with a higher level of MHL reported a lower level of self-stigmatization related to seeking for professional mental health help.

Independent sample t-tests were conducted to examine the differences in MHLS and SSOSH scores between general and vocational secondary school students. The analysis showed that participants who attended general grammar school had a significantly higher score on mental health literacy (*M* = 126.55; *SD* = 11.35) than vocational school students (*M* = 117.87; *SD* = 11.42) with *t(248)* = 5.56; *p* ≤ 0.01; *mean difference* = 8.68; *95%CI* = 5.60–11.75; *d* = 0.71. At the same time, students of general grammar school appeared to experience a lower level of self-stigma related to seeking professional help (*M* = 21.49; *SD* = 5.15) than students of vocational school (*M* = 24.88; *SD* = 5.94) with *t(248)* = −4.33; *p* ≤ 0.01; *mean difference* = 3.39; *95%CI* = (−4.93) – (−1.84); *d* = 1.28. The highest scores in MHLS and the lowest scores in SSOSH were observed in the group of general grammar school students who attended psychology courses as part of their secondary school curriculum (see Table 1). Students in general grammar school who attended psychology classes (*n* = 30) had significantly higher scores in MHLS (*U* = 987; *p* = 0.003, *d* = 0.71) and significantly lower scores in SSOSH (*U* = 498; *p* = 0.031, *d* = 0.51) than their counterparts who had no psychology classes (*n* = 47).

**Table 1:** Descriptive statistics of SSOSH and MHLS scores in subsamples.

School type	Attended psychology course	SSOSH *Mean*	SSOSH *SD*	MHLS *Mean*	MHLS *SD*	*N*
Grammar	Yes	19.27	4.09	129.63	11.36	30
	No	22.91	5.29	124.57	11.01	47
	**Total**	**21.49**	**5.15**	**126.55**	**11.35**	**77**
Vocational	Yes	25.10	5.98	117.77	11.48	162
	No	21.55	4.23	119.36	10.82	11
	**Total**	**24.88**	**5.94**	**117.87**	**11.42**	**173**
Total	Yes	24.19	6.10	119.62	12.22	192
	No	22.66	5.10	123.59	11.07	58
	**Total**	**23.84**	**5.91**	**120.54**	**12.06**	**250**

## 4 Discussion

This brief report aimed to examine the levels of mental health literacy and self-stigma related to help-seeking behavior in Slovak secondary school students. Both instruments were used as unidimensional scales on the recommendation of the authors (11, 27) and appeared to have adequate internal consistency; however, for future research we suggest analyzing the factor structure of both instruments. The findings showed that the mean scores for MHLS and SSOSH in our sample are comparable to those reported by the authors of the original scales for general student populations (27). We found significant differences in the results of the groups of general and vocational secondary school students. Grammar school students (general secondary education) appeared to have higher scores in MHLS and lower scores in SSOSH. In vocational education, the focus is often on acquiring practical skills, and metacognitive competencies, critical thinking, or other literacies tend to be neglected (24, 25). One possible interpretation is that vocational school students may also be more prone to bias and stereotyping in the topic of mental health; however, it needs further exploration.

Help-seeking efficacy is a fundamental part of mental health literacy (28) and, for the improvement of mental health, it is often crucial to access appropriate care (29). Our results support the hypothesis that students with higher scores MHL reported being less biased and stigmatized when seeking professional help, and similar results were found in a sample of university students (30). Therefore, people with high levels of MHL may tend to actively seek social support and professional help, leading to better mental health, while those with lower levels of HML may be reluctant to seek help or delay or avoid seeking help for their mental health problems (29).

Adolescents in Slovakia reported higher levels of stress, anxiety, or symptoms of depression, especially during and after the COVID-19 pandemic (15, 31). However, the availability of mental health services is limited (32), having a school psychologist and other mental health professionals in schools is still not a common standard in Slovak schools. Young people also have negative experiences with stigmatization and that prevents some of them from seeking for professional help (33). Therefore, it is highly relevant to discuss mental health education in schools in Slovakia. Based on our results and previous research findings, it is advisable to provide secondary schools, especially those providing vocational training, with education-based MHL interventions focusing on positive MHL, which are likely to be effective for improving MHL among adolescents (22, 23, 34).

Although the research brought new findings, it has several limitations. The data on the experience of psychology teaching were not adequately distributed, which did not allow us to perform further analysis with relevant statistical power. Due to the relatively small number of participants in the subgroups, we cannot generalize the findings; however, this distribution illustrates the situation in psychology teaching in secondary education in Slovakia. This report is a first exploration of the problem; we did not explore other variables, including sociodemographic factors or previous experience with mental health services. For future research, it is advisable to include those variables, collect the data from an international sample to be able to analyze these trends in a cross-cultural comparison, implement a longitudinal and mixed-method approach to explore the risk factors, and triangulate the data.

## 5 Conclusion

The findings of this brief report suggest that higher levels of mental health literacy may support help seeking efficacy. Due to the fact that vocational school students had significantly lower scores in MHL and higher scores in self-stigma of seeking help, education-based interventions in MHL should be primarily aimed at the population of vocational students; however, these interventions might be beneficial for all secondary school students.

## Data availability statement

The raw data supporting the conclusions of this article will be made available by the authors, without undue reservation.

## Ethics statement

The study was conducted in accordance with the local legislation, institutional requirements, and the Declaration of Helsinki. The participants provided their written informed consent to participate in this study.

## Author contributions

LS: Writing – original draft, Writing – review & editing.
